# *catena*-Poly[di-μ_3_-bromido-hexa-μ_2_-bromido-dibromidobis(*O*-methyl pyridine-2-carboximidate-κ^2^ *N*,*N*′)penta­mercury(II)]

**DOI:** 10.1107/S1600536812046545

**Published:** 2012-11-17

**Authors:** Sadif A. Shirvan, Moayad Hossaini Sadr

**Affiliations:** a Department of Chemistry, Omidieh Branch, Islamic Azad University, Omidieh, Iran; b Department of Chemistry, Faculty of Science, Azarbaijan Shahid Madani University, Tabriz, Iran

## Abstract

The title compound, [Hg_5_Br_10_(C_7_H_8_N_2_O)_2_]_*n*_, contains two μ_3_-bridging Br atoms, six μ_2_-bridging Br atoms and two terminal Br atoms. One Hg^II^ atom, lying on an inversion center, adopts a distorted octa­hedral geometry defined by six Br atoms. Two Hg^II^ atoms adopt a distorted square-pyramidal geometry by five Br atoms and the other two Hg^II^ atoms have a distorted tetra­hedral geometry by two N atoms from a chelating *O*-methyl pyridine-2-carboximidate ligand and two Br atoms. The Br atoms link the Hg^II^ atoms into a ribbon structure along [100].

## Related literature

For metal complexes with *O*-alkyl pyridine-2-carboximidate, see: Barnard (1969 ▶); Du *et al.* (2005 ▶, 2006 ▶); Jamnicky *et al.* (1995 ▶); Seglá & Jamnicky (1988 ▶); Suzuki *et al.* (1974 ▶).
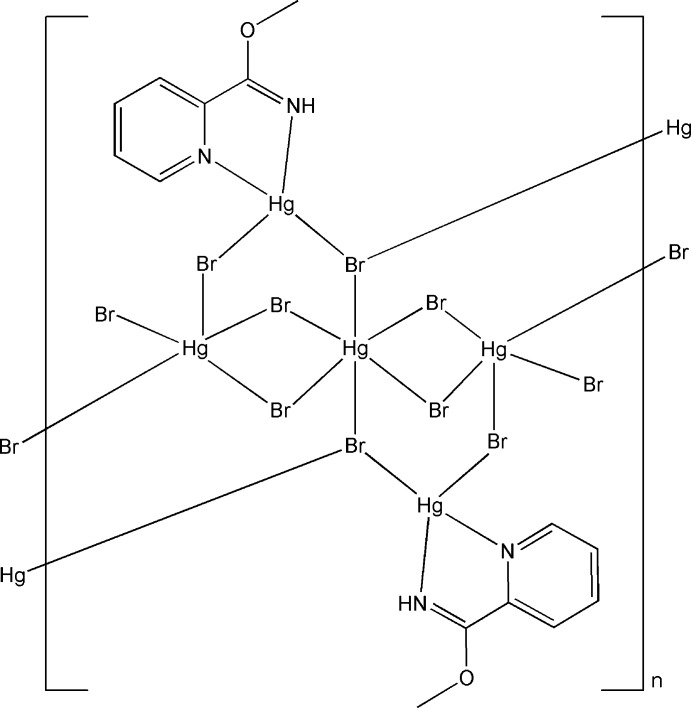


## Experimental

### 

#### Crystal data

[Hg_5_Br_10_(C_7_H_8_N_2_O)_2_]
*M* *_r_* = 2074.26
Triclinic, 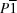

*a* = 7.6768 (7) Å
*b* = 10.7223 (10) Å
*c* = 11.0663 (10) Å
α = 92.067 (7)°
β = 102.850 (7)°
γ = 101.879 (7)°
*V* = 865.86 (14) Å^3^
*Z* = 1
Mo *K*α radiation
μ = 33.65 mm^−1^
*T* = 298 K
0.30 × 0.19 × 0.18 mm

#### Data collection

Bruker APEXII CCD diffractometer
Absorption correction: multi-scan (*SADABS*; Bruker, 2001 ▶) *T*_min_ = 0.105, *T*_max_ = 0.223
7320 measured reflections
3410 independent reflections
2042 reflections with *I* > 2σ(*I*)
*R*_int_ = 0.084

#### Refinement

*R*[*F* ^2^ > 2σ(*F* ^2^)] = 0.062
*wR*(*F* ^2^) = 0.148
*S* = 0.93
3410 reflections
160 parameters
H-atom parameters constrained
Δρ_max_ = 1.89 e Å^−3^
Δρ_min_ = −1.90 e Å^−3^

### 

Data collection: *APEX2* (Bruker, 2007 ▶); cell refinement: *SAINT* (Bruker, 2007 ▶); data reduction: *SAINT*; program(s) used to solve structure: *SHELXS97* (Sheldrick, 2008 ▶); program(s) used to refine structure: *SHELXL97* (Sheldrick, 2008 ▶); molecular graphics: *ORTEP-3* (Farrugia, 2012 ▶); software used to prepare material for publication: *SHELXL97*.

## Supplementary Material

Crystal structure: contains datablock(s) I, global. DOI: 10.1107/S1600536812046545/hy2602sup1.cif

Structure factors: contains datablock(s) I. DOI: 10.1107/S1600536812046545/hy2602Isup2.hkl

Additional supplementary materials:  crystallographic information; 3D view; checkCIF report

## Figures and Tables

**Table 1:** Selected bond lengths (Å)

Hg1—N1	2.322 (14)
Hg1—N2	2.400 (16)
Hg1—Br1	2.524 (2)
Hg1—Br2	2.534 (2)
Hg2—Br2	3.204 (2)
Hg2—Br3	2.438 (2)
Hg2—Br5	3.189 (2)
Hg3—Br1	3.119 (2)
Hg3—Br2^i^	3.140 (2)
Hg3—Br3	3.337 (2)
Hg3—Br4	2.418 (2)
Hg3—Br5	2.463 (2)

Symmetry code: (i) 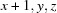.

**Fig. 1.**
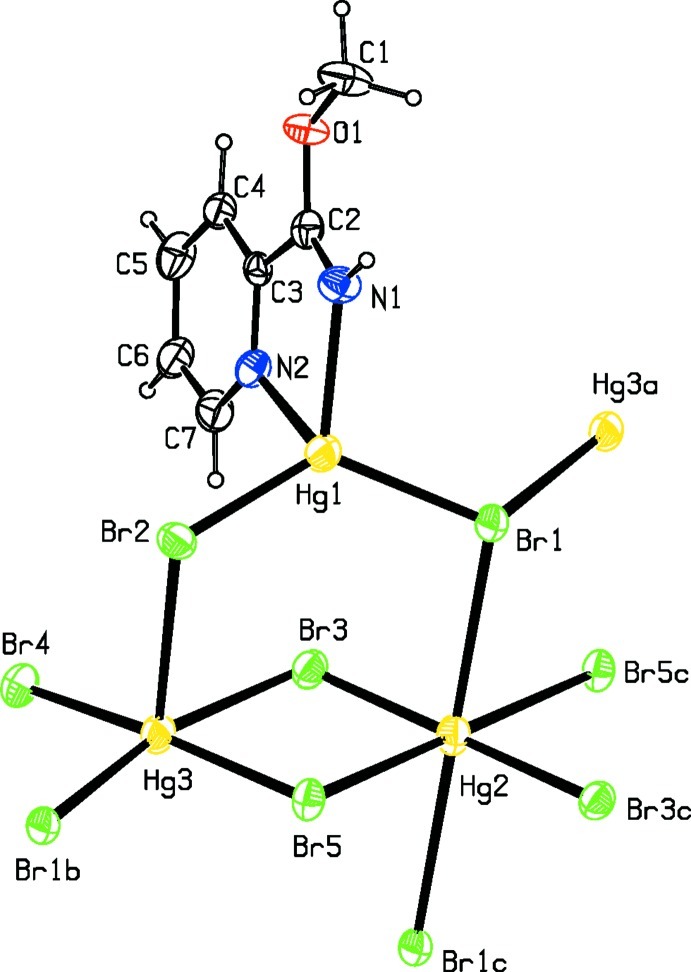
The asymmetric unit of the title compound. Displacement ellipsoids are drawn at the 50% probability level. [Symmetry codes: (a) -1+x, y, z; (b) 1+x, y, z; (c) 1-x, 1-y, -z.]

**Fig. 2.**
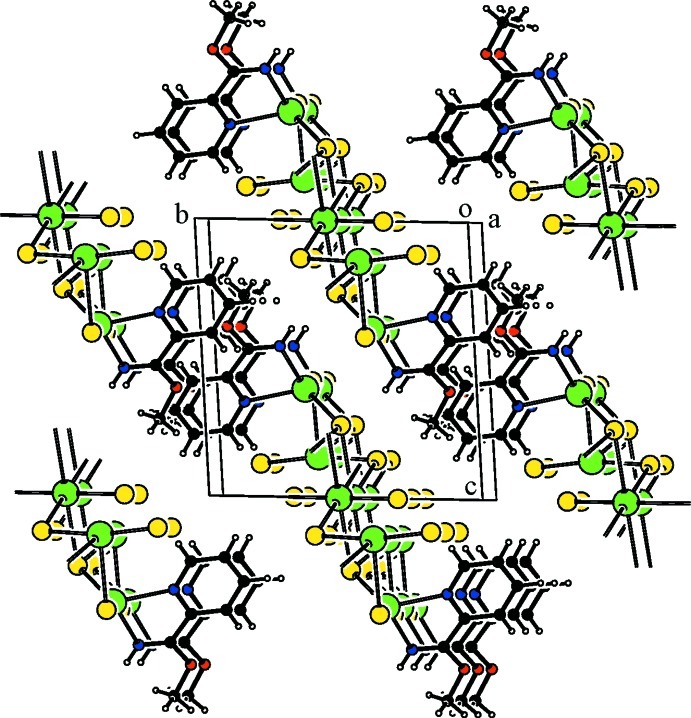
Crystal packing diagram for the title compound.

## supplementary crystallographic information

### Comment

As has previously been observed, the reaction of 2-cyanopyridine (2-cnpy) with water or an alcohol in the presence of some metal(II) salts leads to the formation of complexes which contain *O*-alkylpyridine-2-carboximidate (Barnard, 1969; Seglá & Jamnicky, 1988; Suzuki *et al.*, 1974). The reaction of 2-cnpy and Cu^II^ (Du *et al.*, 2005), Cd^II^ (Du *et al.*, 2006) and Ni^II^ (Jamnicky *et al.*, 1995) in methanol leads to the formation of a chelate ligand, *O*-methylpyridine-2-carboximidate. Here, we report the synthesis and structure of the title compound.

The asymmetric unit of the title compound (Fig. 1) consists of 2.5 crystallographically independent Hg^II^ atoms, five bromide ions and one neutral *O*-methylpyridine-2-carboximidate ligand. The Br2 atom adopts a µ_3_-mode to bridge three Hg^II^ atoms, while Br1, Br3 and Br5 are coordinated to Hg^II^ atoms in a µ_2_-mode. The Br4 atom is coordinated to one Hg^II^ atom in a terminal fashion. The Hg1 atom adopts a distorted tetrahedral coordination geometry defined by two N atoms from one *O*-methylpyridine-2-carboximidate ligand and two Br atoms. The Hg2 atom, lying on an inversion center, adopts a distorted octahedral geometry by six Br atoms and Hg3 atom adopts a distorted square-pyramidal geometry by five Br atoms (Table 1). The Hg^II^ atoms are linked by the Br atoms into a ribbon structure along [100].

### Experimental

For the preparation of the title compound, a solution of 2-cyanopyridine (0.47 g, 4.42 mmol) in methanol (10 ml) was added to a solution of HgBr_2_ (0.82 g, 2.21 mmol) in methanol (10 ml) and the resulting yellow solution was stirred for 20 min at room temperature. This solution was left to evaporate slowly at room temperature. After one week, yellow prismatic crystals of the title compound were isolated (yield: 0.75 g, 83.4%).

### Refinement

All H atoms were positioned geometrically and refined as riding atoms, with C—H = 0.93 (aromatic), 0.96 (CH_3_) and N—H = 0.86 Å and with *U*_iso_(H) = 1.2*U*_eq_(C, N). The highest residual electron density was found 1.09 Å from Hg3 the deepest hole 1.05 Å from Hg3.

### Crystal data

**Table:**

[Hg_5_Br_10_(C_7_H_8_N_2_O)_2_]	*Z* = 1
*M**_r_* = 2074.26	*F*(000) = 894
Triclinic, *P*1	*D*_x_ = 3.978 Mg m^−^^3^
Hall symbol: -P 1	Mo *K*α radiation, λ = 0.71073 Å
*a* = 7.6768 (7) Å	Cell parameters from 7320 reflections
*b* = 10.7223 (10) Å	θ = 1.9–26.0°
*c* = 11.0663 (10) Å	µ = 33.65 mm^−^^1^
α = 92.067 (7)°	*T* = 298 K
β = 102.850 (7)°	Prism, yellow
γ = 101.879 (7)°	0.30 × 0.19 × 0.18 mm
*V* = 865.86 (14) Å^3^	

### Data collection

**Table:**

Bruker APEXII CCD diffractometer	3410 independent reflections
Radiation source: fine-focus sealed tube	2042 reflections with *I* > 2σ(*I*)
Graphite monochromator	*R*_int_ = 0.084
φ and ω scans	θ_max_ = 26.0°, θ_min_ = 1.9°
Absorption correction: multi-scan (*SADABS*; Bruker, 2001)	*h* = −9→9
*T*_min_ = 0.105, *T*_max_ = 0.223	*k* = −13→13
7320 measured reflections	*l* = −13→13

### Refinement

**Table:**

Refinement on *F*^2^	Primary atom site location: structure-invariant direct methods
Least-squares matrix: full	Secondary atom site location: difference Fourier map
*R*[*F*^2^ > 2σ(*F*^2^)] = 0.062	Hydrogen site location: inferred from neighbouring sites
*wR*(*F*^2^) = 0.148	H-atom parameters constrained
*S* = 0.93	*w* = 1/[σ^2^(*F*_o_^2^) + (0.0731*P*)^2^] where *P* = (*F*_o_^2^ + 2*F*_c_^2^)/3
3410 reflections	(Δ/σ)_max_ = 0.003
160 parameters	Δρ_max_ = 1.89 e Å^−^^3^
0 restraints	Δρ_min_ = −1.90 e Å^−^^3^

### Special details

**Table:**

Geometry. All e.s.d.'s (except the e.s.d. in the dihedral angle between two l.s. planes) are estimated using the full covariance matrix. The cell e.s.d.'s are taken into account individually in the estimation of e.s.d.'s in distances, angles and torsion angles; correlations between e.s.d.'s in cell parameters are only used when they are defined by crystal symmetry. An approximate (isotropic) treatment of cell e.s.d.'s is used for estimating e.s.d.'s involving l.s. planes.
Refinement. Refinement of *F*^2^ against ALL reflections. The weighted *R*-factor *wR* and goodness of fit *S* are based on *F*^2^, conventional *R*-factors *R* are based on *F*, with *F* set to zero for negative *F*^2^. The threshold expression of *F*^2^ > σ(*F*^2^) is used only for calculating *R*-factors(gt) *etc*. and is not relevant to the choice of reflections for refinement. *R*-factors based on *F*^2^ are statistically about twice as large as those based on *F*, and *R*- factors based on ALL data will be even larger.

### Fractional atomic coordinates and isotropic or equivalent isotropic displacement parameters (Å^2^)

**Table:**

	*x*	*y*	*z*	*U*_iso_*/*U*_eq_	
C1	−0.003 (4)	0.838 (2)	−0.726 (2)	0.076 (7)	
H1A	−0.0886	0.7662	−0.7095	0.091*	
H1B	0.0734	0.8091	−0.7743	0.091*	
H1C	−0.0680	0.8955	−0.7723	0.091*	
C2	0.211 (2)	0.8446 (18)	−0.5315 (18)	0.046 (4)	
C3	0.327 (3)	0.9261 (17)	−0.4289 (17)	0.046 (4)	
C4	0.329 (3)	1.0579 (19)	−0.4102 (19)	0.056 (5)	
H4	0.2567	1.0962	−0.4702	0.067*	
C5	0.434 (3)	1.128 (2)	−0.3060 (19)	0.057 (5)	
H5	0.4316	1.2142	−0.2930	0.069*	
C6	0.540 (3)	1.075 (2)	−0.223 (2)	0.063 (6)	
H6	0.6140	1.1230	−0.1517	0.076*	
C7	0.539 (3)	0.948 (2)	−0.244 (2)	0.065 (6)	
H7	0.6186	0.9131	−0.1855	0.078*	
N1	0.211 (2)	0.7237 (14)	−0.5445 (16)	0.054 (4)	
H1D	0.1406	0.6742	−0.6067	0.065*	
N2	0.435 (3)	0.8714 (16)	−0.3409 (15)	0.059 (5)	
O1	0.110 (2)	0.9040 (13)	−0.6109 (12)	0.062 (4)	
Hg1	0.41370 (13)	0.64830 (8)	−0.39109 (9)	0.0623 (3)	
Hg2	0.5000	0.5000	0.0000	0.0588 (3)	
Hg3	0.95464 (12)	0.64042 (7)	−0.14661 (8)	0.0591 (3)	
Br1	0.7283 (3)	0.6379 (2)	−0.4186 (2)	0.0619 (6)	
Br2	0.2253 (3)	0.5162 (2)	−0.26046 (19)	0.0549 (5)	
Br3	0.6541 (3)	0.72282 (19)	−0.0001 (2)	0.0607 (5)	
Br4	1.1178 (4)	0.8617 (2)	−0.1174 (3)	0.0788 (7)	
Br5	0.7893 (3)	0.41969 (18)	−0.1371 (2)	0.0544 (5)	

### Atomic displacement parameters (Å^2^)

**Table:**

	*U*^11^	*U*^22^	*U*^33^	*U*^12^	*U*^13^	*U*^23^
C1	0.070 (15)	0.080 (16)	0.076 (17)	0.026 (13)	0.004 (13)	0.002 (13)
C2	0.027 (9)	0.057 (11)	0.051 (12)	0.002 (8)	0.008 (8)	0.012 (9)
C3	0.049 (10)	0.043 (9)	0.038 (10)	−0.010 (8)	0.011 (9)	0.005 (8)
C4	0.057 (12)	0.055 (12)	0.051 (13)	0.007 (10)	0.007 (10)	−0.001 (10)
C5	0.061 (13)	0.053 (11)	0.052 (12)	0.001 (10)	0.016 (11)	−0.018 (9)
C6	0.058 (13)	0.058 (13)	0.072 (15)	−0.002 (11)	0.028 (12)	−0.011 (11)
C7	0.053 (13)	0.065 (14)	0.058 (14)	−0.023 (11)	0.006 (11)	0.007 (11)
N1	0.059 (10)	0.035 (8)	0.068 (11)	0.019 (8)	0.008 (9)	0.007 (7)
N2	0.078 (12)	0.051 (9)	0.038 (9)	0.015 (9)	−0.008 (9)	0.003 (7)
O1	0.078 (10)	0.055 (8)	0.047 (8)	0.030 (8)	−0.010 (7)	−0.003 (6)
Hg1	0.0624 (5)	0.0600 (5)	0.0714 (6)	0.0192 (4)	0.0229 (4)	0.0187 (4)
Hg2	0.0572 (7)	0.0560 (6)	0.0657 (8)	0.0076 (5)	0.0246 (6)	0.0032 (5)
Hg3	0.0575 (5)	0.0477 (4)	0.0686 (6)	0.0021 (4)	0.0159 (4)	0.0045 (4)
Br1	0.0548 (12)	0.0756 (14)	0.0564 (13)	0.0195 (11)	0.0106 (10)	0.0052 (10)
Br2	0.0528 (11)	0.0612 (12)	0.0505 (12)	0.0070 (9)	0.0162 (9)	0.0068 (9)
Br3	0.0698 (14)	0.0512 (11)	0.0631 (13)	0.0113 (10)	0.0208 (11)	0.0071 (9)
Br4	0.0762 (16)	0.0521 (12)	0.0983 (19)	−0.0031 (11)	0.0148 (14)	0.0127 (12)
Br5	0.0573 (12)	0.0446 (10)	0.0632 (13)	0.0040 (9)	0.0263 (10)	−0.0037 (9)

### Geometric parameters (Å, º)

**Table:**

C1—O1	1.44 (3)	C7—N2	1.32 (3)
C1—H1A	0.9600	C7—H7	0.9300
C1—H1B	0.9600	Hg1—N1	2.322 (14)
C1—H1C	0.9600	N1—H1D	0.8600
C2—N1	1.30 (2)	Hg1—N2	2.400 (16)
C2—O1	1.313 (19)	Hg1—Br1	2.524 (2)
C2—C3	1.42 (3)	Hg1—Br2	2.534 (2)
C3—N2	1.36 (2)	Hg2—Br2	3.204 (2)
C3—C4	1.42 (3)	Hg2—Br3	2.438 (2)
C4—C5	1.35 (3)	Hg2—Br5	3.189 (2)
C4—H4	0.9300	Hg3—Br1	3.119 (2)
C5—C6	1.31 (3)	Hg3—Br2^i^	3.140 (2)
C5—H5	0.9300	Hg3—Br3	3.337 (2)
C6—C7	1.38 (3)	Hg3—Br4	2.418 (2)
C6—H6	0.9300	Hg3—Br5	2.463 (2)
			
O1—C1—H1A	109.5	N2—C7—C6	125 (2)
O1—C1—H1B	109.5	N2—C7—H7	117.5
H1A—C1—H1B	109.5	C6—C7—H7	117.5
O1—C1—H1C	109.5	C2—N1—Hg1	116.5 (13)
H1A—C1—H1C	109.5	C2—N1—H1D	121.7
H1B—C1—H1C	109.5	Hg1—N1—H1D	121.7
N1—C2—O1	124.8 (18)	C7—N2—C3	116.5 (17)
N1—C2—C3	121.8 (16)	C7—N2—Hg1	129.5 (14)
O1—C2—C3	113.4 (17)	C3—N2—Hg1	113.9 (12)
N2—C3—C2	117.1 (16)	C2—O1—C1	120.6 (16)
N2—C3—C4	119.2 (17)	N1—Hg1—N2	70.5 (5)
C2—C3—C4	123.6 (17)	N1—Hg1—Br1	120.2 (4)
C5—C4—C3	120.6 (18)	N2—Hg1—Br1	104.2 (5)
C5—C4—H4	119.7	N1—Hg1—Br2	107.2 (4)
C3—C4—H4	119.7	N2—Hg1—Br2	109.5 (5)
C6—C5—C4	120 (2)	Br1—Hg1—Br2	128.56 (7)
C6—C5—H5	120.2	Br3^ii^—Hg2—Br3	180.0
C4—C5—H5	120.2	Br4—Hg3—Br5	169.89 (9)
C5—C6—C7	119 (2)	Br4—Hg3—Br1	98.67 (8)
C5—C6—H6	120.5	Br5—Hg3—Br1	89.75 (7)
C7—C6—H6	120.5	Hg1—Br1—Hg3	103.35 (8)
			
N1—C2—C3—N2	−3 (3)	N1—C2—O1—C1	−4 (3)
O1—C2—C3—N2	179.0 (17)	C3—C2—O1—C1	173.9 (19)
N1—C2—C3—C4	−179.0 (19)	C2—N1—Hg1—N2	1.7 (14)
O1—C2—C3—C4	3 (3)	C2—N1—Hg1—Br1	−93.8 (14)
N2—C3—C4—C5	−1 (3)	C2—N1—Hg1—Br2	106.8 (14)
C2—C3—C4—C5	176 (2)	C7—N2—Hg1—N1	180 (2)
C3—C4—C5—C6	2 (3)	C3—N2—Hg1—N1	−3.0 (13)
C4—C5—C6—C7	−1 (3)	C7—N2—Hg1—Br1	−63 (2)
C5—C6—C7—N2	−2 (4)	C3—N2—Hg1—Br1	114.5 (14)
O1—C2—N1—Hg1	177.9 (14)	C7—N2—Hg1—Br2	78 (2)
C3—C2—N1—Hg1	0 (2)	C3—N2—Hg1—Br2	−104.9 (14)
C6—C7—N2—C3	3 (3)	N1—Hg1—Br1—Hg3	157.3 (5)
C6—C7—N2—Hg1	−179.3 (17)	N2—Hg1—Br1—Hg3	81.8 (4)
C2—C3—N2—C7	−178.4 (19)	Br2—Hg1—Br1—Hg3	−48.13 (13)
C4—C3—N2—C7	−2 (3)	Br4—Hg3—Br1—Hg1	−102.16 (10)
C2—C3—N2—Hg1	4 (2)	Br5—Hg3—Br1—Hg1	72.22 (8)
C4—C3—N2—Hg1	−179.6 (15)		

Symmetry codes: (i) *x*+1, *y*, *z*; (ii) −*x*+1, −*y*+1, −*z*.
